# A nutrient-sensing protease DegS suppresses biofilm formation in *Vibrio cholerae* via the CdgH–c-di-GMP–VpsR axis under nutrient limitation

**DOI:** 10.1016/j.bioflm.2026.100398

**Published:** 2026-09-10

**Authors:** Xiaoyu Huang, Tao Chen, Qiqi Linghu, Hui Wang, Shilu Luo, Huan Zhou, Xun Min, Jian Huang

**Affiliations:** a Department of Laboratory Medicine, Affiliated Hospital of Zunyi Medical University, Zunyi, 563000, China; b School of Laboratory Medicine, Zunyi Medical University, Zunyi, 563000, China

**Keywords:** *Vibrio cholerae*, DegS protease, Biofilm, CdgH–c-di-GMP–VpsR axis, Nutrient limitation

## Abstract

The transition of *Vibrio cholerae* from the host intestine to oligotrophic aquatic environments presents a severe nutrient downshift, yet the upstream sensory mechanism that triggers adaptive biofilm formation remains unclear. Here, we identify the periplasmic protease DegS as a critical nutrient-responsive regulator that suppresses biofilm development under low-nutrient conditions. We demonstrate that a *ΔdegS* mutant exhibits robust biofilm formation, enhanced antibiotic tolerance, and significantly increased colonization on environmentally relevant surfaces such as microplastics. Strikingly, this regulation is independent of the canonical σ^E^ stress pathway. Instead, we elucidate a novel signalling axis wherein DegS negatively regulates the diguanylate cyclase CdgH. Loss of DegS relieves this inhibition, leading to elevated cellular c-di-GMP levels, which in turn activates the master transcriptional regulator VpsR, upregulating biofilm matrix gene expression. This DegS-CdgH-c-di-GMP-VpsR pathway is functional not only in minimal medium but also in simulated natural aquatic environments. Our findings reveal DegS as a key upstream sensor that translates nutrient scarcity into a precise inhibitory signal via c-di-GMP-dependent transcription, providing new insights into the molecular basis of *V. cholerae* environmental adaptation and highlighting a potential target within its transmission chain.

## Highlights

• DegS is a periplasmic nutrient sensor that suppresses *V. cholerae* biofilm under nutrient limitation.
• A novel DegS–CdgH–c-di-GMP–VpsR axis links nutrient scarcity to biofilm transcriptional control.
• This signaling pathway operates in natural aquatic environments and on microplastic surfaces.

## 1 Introduction

*Vibrio cholerae* is the causative agent of cholera, a severe acute secretory diarrhea disease characterised by rapid fluid loss that can lead to profound dehydration and death [1]. The transmission of *V. cholerae* follows a defined fecal-oral route, driven by a life cycle that alternates sharply between two disparate environments: the nutrient-rich lumen of the human small intestine and oligotrophic aquatic reservoirs [2]. Upon ingestion of contaminated water, the bacterium colonizes the intestinal epithelium, leading to the profuse production of characteristic "rice-water" stools that release vast numbers of cells back into the environment [3]. This exit presents a critical physiological challenge: the bacterium must rapidly adapt from a nutrient-replete host milieu to a nutrient-poor aquatic state to await new opportunities for infection [4].

In these austere environmental niches, the formation of structured, surface-associated communities known as biofilms represents a cornerstone survival strategy. Biofilms confer enhanced resistance to diverse stresses, including osmotic fluctuation, predation, and antibiotics, thereby functioning as a durable environmental reservoir that ensures bacterial persistence between outbreaks and facilitates geographic dissemination [5]. Conversely, within the host, biofilm-like aggregates have been shown to significantly enhance intestinal colonization and provide protection against host immune clearance through a physical barrier effect. Thus, biofilm formation plays an indispensable role in both key phases of the *V. cholerae* transmission cycle: efficient host colonization and long-term environmental survival [6].

The formation and dispersal of *V. cholerae* biofilms are dynamically regulated by a complex network that integrates the second messenger cyclic diguanylate (c-di-GMP), quorum-sensing systems, and key transcriptional regulators such as VpsR and VpsT, coordinating the transition between motile and sessile lifestyles [7,8]. The shift from the host to the environment is accompanied by drastic nutritional downshift, and the bacterium senses and responds to specific nutrient cues to modulate biofilm behavior. For instance, carbon availability influences biofilm formation via pathways including the phosphoenolpyruvate phosphotransferase system (PTS) [9]; phosphate limitation regulates biofilm gene expression through the PhoBR two-component system [10]; and iron availability modulates c-di-GMP metabolism and matrix production via the Fur protein and the small RNA RyhB [11]. These findings establish that various specific nutrient signals can be integrated into the downstream biofilm regulatory network through defined molecular pathways.

However, despite this progressive elucidation of downstream integration mechanisms, a more fundamental question remains unresolved: What category of upstream sensory components first detects the comprehensive and fundamental decrease in environmental nutrient levels when *V. cholerae* transitions from the host into an oligotrophic aquatic environment? Furthermore, how is this critical survival stress signal subsequently transduced and relayed to generate the precise instruction that initiates the biofilm developmental program? The identity and operational logic of these primary sensory and signal transduction elements constitute a central gap in understanding *V. cholerae's* environmental adaptation cycle. Deciphering this initial detection step is essential for a complete comprehension of its environmental persistence and for developing targeted intervention strategies.

The periplasmic serine protease DegS is a key regulator in the σ^E^ (*rpoE*) stress response pathway [12]. Previous studies have demonstrated that in *V. cholerae*, DegS is involved in regulating various physiological processes, including motility, the oxidative stress response, and energy metabolism [[13], [14], [15]]. Nevertheless, its potential role in modulating collective behaviors such as biofilm formation has remained unexplored. In this study, we identify DegS as a critical inhibitory regulator of biofilm development under low-nutrient conditions. We show that a *ΔdegS* mutant exhibits significantly enhanced biofilm formation, increased antibiotic tolerance, and a markedly improved ability to colonize environmentally relevant surfaces such as microplastics. Mechanistically, we elucidate a previously unrecognized signalling axis in which DegS, functioning independently of the canonical σ^E^ pathway, negatively regulates the diguanylate cyclase CdgH. This inhibition leads to elevated cellular c-di-GMP levels, which in turn activates the transcriptional regulator VpsR and upregulates the expression of biofilm matrix genes. This DegS–CdgH–c-di-GMP–VpsR signalling pathway is functional not only in defined minimal medium but also in simulated natural aquatic environments, underscoring its ecological relevance. Our findings reveal a novel regulatory circuit that functionally links a periplasmic protease to c-di-GMP-dependent transcriptional control, providing new insights into the molecular basis of *V. cholerae* environmental adaptation and highlighting a potential target within its transmission chain.

## 2 Materials and methods

### 2.1 Bacterial strains and growth conditions

Non-O1/non-O139 *V. cholerae* HN375 (CCTCCAB209168) was used as wild-type (WT) [16]. For cloning experiments, *Escherichia coli* DH5α and DH5α-λpir were employed, while *E. coli* WM3064 served as the donor strain in conjugation assays. *V. cholerae* strains were routinely cultured in lysogeny broth (LB) (10 g/L tryptone, 5 g/L yeast extract, 10 g/L NaCl) or M9 minimal medium (1× M9 salts, 2 mM MgSO_4_, 100 μM CaCl_2_, 0.8% glucose) at 37°C with shaking at 200 rpm. *E. coli* strains were grown in LB at 37°C with shaking at 200 rpm. When required, antibiotics were used at the following concentrations: ampicillin (100 μg/mL) and spectinomycin (100 μg/mL). For induction of gene expression from the pBAD24-derived plasmids, 0.1% (w/v) l-arabinose was added to the medium where indicated. Detailed information for all plasmids and bacterial strains used in this study is provided in Supplementary Table 1. For nutrient gradient experiments, M9 minimal medium was prepared at 0.5×, 1×, and 2× concentrations by diluting all components proportionally.

### 2.2 DNA manipulations and genetic techniques

Gene deletion mutants from the WT HN375 strain were constructed using the suicide plasmid pWM91 [17]. Primer sequences are listed in Supplementary Table 2. To construct complementary strains, the full-length coding sequences of *vpsR* and *cdgH* were individually cloned into the pBAD24 vector. The resulting recombinant plasmids were then introduced via conjugation into the *ΔdegSΔvpsR* and *ΔdegSΔcdgH* mutants, respectively, generating the complementary strains *ΔdegSΔvpsR::vpsR* and *ΔdegSΔcdgH::cdgH*. [18]. All complemented strains were grown in medium containing 0.1% arabinose for gene expression induction. To control for any effects of the pBAD24 vector backbone itself, the empty plasmid was introduced into the mutant strains.

### 2.3 Recombinant protein expression, purification, and polyclonal antiserum preparation

The coding regions of *vpsR* and *cdgH* were amplified and cloned into the plasmid pCold-TF (Takara). These recombinant plasmids were transformed into *E. coli* BL21 strain for protein expression. The His-tagged recombinant proteins were purified using Ni^2+^-NTA affinity chromatography. To prepare anti-VpsR and anti-CdgH antisera for Western blotting, 6-week-old BALB/c mice were randomly assigned to groups (n = 10). Mice were immunized subcutaneously with 30 μg of recombinant VpsR or CdgH protein mixed with an equal volume of aluminum adjuvant on days 1, 14, and 28. Blood samples were collected from mouse eyes on day 42, and serum titers were determined by ELISA [19]. All animal experiments were approved by the Ethics Committee of Zunyi Medical University (No. ZMU21-2301-069).

### 2.4 Western blot analysis

All strains were inoculated into liquid LB medium and grown at 37°C with shaking at 200 rpm until OD_600_ reached 0.6. Cultures were diluted 100-fold in M9 medium, and 2 mL aliquots were added to sterile 12-well plates. Plates were incubated statically at 37°C in a 5% CO_2_ incubator for 24 h. Biofilm cells were dislodged by pipetting, collected by centrifugation, and resuspended in 1× SDS-PAGE loading buffer. Samples were boiled for 10 min and stored at 4°C until use. Proteins were separated by SDS-PAGE on 10% polyacrylamide gels at 25–30 mA/gel under a maximum voltage of 120 V. Following electrophoresis, gels were equilibrated in transfer buffer containing 10 mM EDTA, followed by EDTA-free transfer buffer, and then transferred onto PVDF membranes. Membranes were blocked with 5% non-fat milk in TBST and incubated with primary antibodies as follows: anti-VpsR (1:1,000), anti-CdgH (1:1,000), or anti-His-tag (Proteintech, mouse monoclonal, 1:2,000). After washing, membranes were incubated with HRP-conjugated goat anti-mouse secondary antibody (Proteintech, 1:5,000). Immunoreactive signals were detected using enhanced chemiluminescence (ECL) substrate. RpoA (BioLegend, 1:2,000) served as the loading control [20]. All experiments were performed in triplicate.

### 2.5 Quantitative RT-PCR

Biofilm cells were collected as described above, and total RNA was extracted using RNAiso Plus (Takara) according to the manufacturer's instructions. Residual genomic DNA was removed by DNase I treatment (Takara). RNA concentration and purity were determined using a NanoDrop spectrophotometer (Thermo Fisher). First-strand cDNA was synthesized from 1 μg of total RNA using a PrimeScript RT reagent kit with gDNA Eraser (Takara). qRT-PCR was performed using TB Green Premix Ex Taq II (Takara) on a Gentier 96E Real-Time PCR System (Tianlong) [21]. The thermal cycling program was: initial denaturation at 95°C for 30 s, followed by 40 cycles of 95°C for 5 s and 60°C for 30 s. Melting curve analysis was performed to verify the specificity of each primer pair. Relative mRNA levels were calculated using the 2^−ΔΔCt^ method, with 16S rRNA as the internal control. Primers used for qRT-PCR are listed in Supplementary Table 2. For each qRT-PCR, three independent experiments were performed, each with three technical replicates. Statistical summary of all qRT-PCR data (mean ± SD, n = 3) is provided in Supplementary Table 3.

### 2.6 Quantitative biofilm assay and bacterial growth curves

Biofilms were inoculated as described above, with 200 μL aliquots added to sterile 96-well U-bottom polystyrene plates (Costar) and incubated statically at 37°C in a 5% CO_2_ incubator. After incubation, to monitor planktonic cell growth and generate growth curves, the optical density at 600 nm (OD_600_) of the culture medium in each well was measured using a microplate reader. Following OD_600_ measurement, the medium was discarded, and the plates were washed three times with sterile PBS. Residual liquid was removed by tapping the plates dry. Next, 200 μL of 0.1% crystal violet (CV) solution was added to each well and incubated for 20 min for staining. The stain was discarded, and the plates were washed three times with sterile PBS to remove unbound crystal violet. Plates were then tapped dry to remove residual liquid. For destaining, 200 μL of absolute ethanol was added to each well and incubated for 20 min. The absorbance at 590 nm (OD_590_) was measured using a microplate reader [22]. Multiple 96-well plates were prepared for biofilm cultivation, with crystal violet (CV) staining performed every 2 h for the first 24 h and every 24 h thereafter for up to 168 h. Three independent experiments were performed, each with three technical replicates.

### 2.7 Scanning electron microscopy (SEM)

Static biofilms were grown on sterile glass coverslips in 12-well plates containing 2 mL M9 medium and incubated at 37°C in a 5% CO_2_ incubator for 48 h. The coverslips were removed, and planktonic cells were gently washed away with PBS. The samples were fixed in 2.5% (v/v) glutaraldehyde in 0.1 M sodium cacodylate buffer (pH 7.4) at 4°C for 12 h, followed by two washes with phosphate-buffered saline for 5 min each. Dehydration was performed using a graded ethanol series (30%, 50%, 70%, 80%, 90%, 95%, and 100%) for 10 min each, followed by treatment with tert-butanol for 15 min. The samples were then lyophilized, mounted on aluminum stubs, and coated with a 10-nm layer of gold-palladium using a sputter coater. Specimens were observed under a field-emission scanning electron microscope (FESEM SU8010, Hitachi, Japan) at an accelerating voltage of 5 kV [11,23]. Images were acquired for three independent replicates.

### 2.8 Biofilms structure analysis

Static biofilms were grown on sterile glass coverslips in 12-well plates as described above for 24 h, and planktonic cells were gently washed away with PBS. The coverslips were fixed in 5% formaldehyde for 30 min. The fixed biofilm samples were stained for 1 h with SYTOX™ Blue (Invitrogen), fluorescein isothiocyanate (FITC, Sigma-Aldrich), and wheat germ agglutinin conjugated to Alexa Fluor 555 (WGA-Alexa Fluor 555, Invitrogen) [24]. Excess stain was removed by gently washing the slides with PBS. Samples were observed using a confocal laser scanning microscope (CLSM, STELLARIS 5, Leica, Germany) with the following settings: SYTOX Blue (excitation 405 nm, emission 421–460 nm), FITC (excitation 488 nm, emission 500–540 nm), and WGA-Alexa Fluor 555 (excitation 561 nm, emission 570–620 nm). Z-stack images were acquired with a step size of 0.5 μm, and three-dimensional projection images were reconstructed using Leica imaging software [25]. Images were acquired for three independent replicates.

### 2.9 Antibiotics killing assay

M9-diluted bacterial cultures were used to inoculate static biofilms in two 96-well plates. After 24 h of incubation, five antibiotics-azithromycin, erythromycin, doxycycline, tetracycline, and ciprofloxacin-were added to the wells of one plate at a final concentration of 64 μg/mL each, while an equivalent volume of the respective solvent (water, ethanol, or DMSO) was added to the other plate as the solvent control. These concentrations were chosen based on preliminary assays as sublethal concentrations that allow biofilm-associated cells to survive while effectively inhibiting planktonic growth. After 6 h of treatment, the medium was removed, and biofilms were washed with PBS. Biofilm cells were detached by vigorous pipetting, serially diluted, and plated onto LB agar plates for colony counting [26]. The survival rate of *V. cholerae* was calculated by dividing the cell count obtained from the antibiotic-treated sample by that from the solvent-treated control. Three independent experiments were performed, each with three technical replicates. To determine whether the enhanced antibiotic tolerance of the *ΔdegS* mutant depends on VpsR-mediated biofilm formation, the same antibiotic killing assay was performed with the *ΔdegSΔvpsR* double mutant, its complemented strain (*ΔdegSΔvpsR::vpsR*), and the empty vector control (*ΔdegSΔvpsR* + pBAD24) under identical conditions.

### 2.10 Microplastics biofilm development assay

Five types of microplastics (MPs) — polyethylene terephthalate (PET), low-density polyethylene (LDPE), high-density polyethylene (HDPE), polypropylene (PP), and polystyrene (PS) — with particle sizes ranging from 50 to 200 μm were used in this study. MPs were sterilized by immersion in 70% ethanol for 30 min, washed three times with sterile distilled water, and air-dried under sterile conditions before use. Each type of MP was added to 50 mL tubes at a concentration of 2 g/L, followed by 20 mL of M9-diluted bacterial cultures. The mixtures were incubated at 37°C with shaking at 200 rpm for 24 h [27]. After incubation, the MP samples were filtered using a sterilized cell strainer (Biosharp, China) and washed three times with PBS to remove planktonic cells. The samples were resuspended in 10 mL of PBS and sonicated at 40 kHz for 10 min to detach biofilm cells. The MPs were removed by filtration, and the bacterial suspensions were serially diluted and plated on LB agar for viable cell counting. The number of adherent cells per gram of microplastic was calculated and expressed as CFU/g MP. Three independent experiments were performed, each with three replicates, and the data were statistically analysed.

### 2.11 c-di-GMP reporter assay

*V. cholerae* strains harboring the c-di-GMP biosensor plasmid pRP0122-Pbe-amcyan_Bc3-5_turborfp were grown in LB medium supplemented with gentamicin (15 μg/mL) to maintain the plasmid [28]. Overnight cultures were diluted 1:100 into M9 medium and used to inoculate static biofilms in 12-well plates at 37°C in a 5% CO_2_ incubator for 24 h. After incubation, 1 mL of bacterial suspension was collected by vigorous pipetting and washed twice with PBS. The samples were diluted to an OD_600_ of 0.1 with PBS and stored at 4°C for subsequent experiments. Fluorescence signals for TurboRFP (excitation 553 nm, emission 593 nm) and AmCyan (excitation 458 nm, emission 498 nm) were measured using a microplate reader (Thermo 3020). The relative fluorescence intensity (RFI) was calculated as the ratio of AmCyan to TurboRFP (AmCyan/TurboRFP), which is inversely proportional to intracellular c-di-GMP levels, with higher RFI indicating lower c-di-GMP concentrations [28,29]. Three independent experiments were performed, each with three technical replicates.

### 2.12 Seawater/Lake water biofilm assay

The biofilm formation capacity of *V. cholerae* was evaluated in artificial seawater (Codow, China) and filter-sterilized lake water (FSLW) [30]. FSLW samples were collected from the artificial lake at the wetland park of Zunyi Medical University’s Xinpu Campus (27°43’22.81”N, 107°4’6.77”E) during the autumn. Water was collected approximately 30 cm from the shore at a depth of 0.5 m using a sterile 1 L conical flask. The water was filter-sterilized using a 0.22-μm-pore-size syringe filter (Jinteng). The chemical and biochemical properties of the unfiltered lake water were analysed by Centre Testing International company [31]. The results are reported as follows (in mg/L): 2.0 total organic carbon, 0.61 total nitrogen, 0.226 NH_4_^+^, 0.02 total phosphorus, 4.0 K, 29.8 Mg, 6.65 Na, 46.0 Ca, 5.10 Cl^−^, 0.216 Al, 0.0299 Ba, 0.00056 Cu, 0.158 Fe, 0.00498 Mn, 20.3 S, 43.4 SO_4_^2−^, 0.62 Sr, and 0.00166 Zn, 0.00102 As, 0.00032 Cr, 0.00072 Ni, and 0.00029 Pb. The levels of Be (<0.00004), Cd (<0.00001), NO_3_^−^ (<0.016), and NO_2_^−^ (<0.016) were below the detection limit. At the time of collection, the water had a pH of 7.6 and a temperature of 14.2°C.

Bacterial strains were inoculated into liquid LB medium and grown at 37°C with shaking at 200 rpm until the OD_600_ reached 0.6. Cells were harvested by centrifugation at 5000 rpm for 5 min, washed twice with either seawater or lake water, and resuspended in the respective water to adjust the bacterial concentration to an OD_600_ of 0.7. Aliquots of 200 μL were added to sterile 96-well plates and incubated statically at 37°C in a 5% CO_2_ incubator for 24 h. Biofilms were stained with crystal violet as described above. Three independent experiments were performed, each with three replicates, and the data were statistically analysed.

### 2.13 Statistical analysis

Data analysis and graphing were performed using GraphPad Prism 9.0 software. Data are presented as mean ± standard deviation (SD) from at least three independent experiments. Two-tailed Student's t-tests were used for comparisons between two groups, and one-way or two-way ANOVA followed by Tukey's post-hoc test for all pair-wise comparisons was used for multiple group comparisons. Statistical significance was defined as *P* < 0.05. Significance levels are indicated as follows: *P < 0.05, **P < 0.01, ***P < 0.001, and ****P < 0.0001; ns indicates not significant.

## 3 Results

### 3.1 DegS suppresses biofilm formation in *V. cholerae* under low-nutrient conditions

**Fig. 1:**
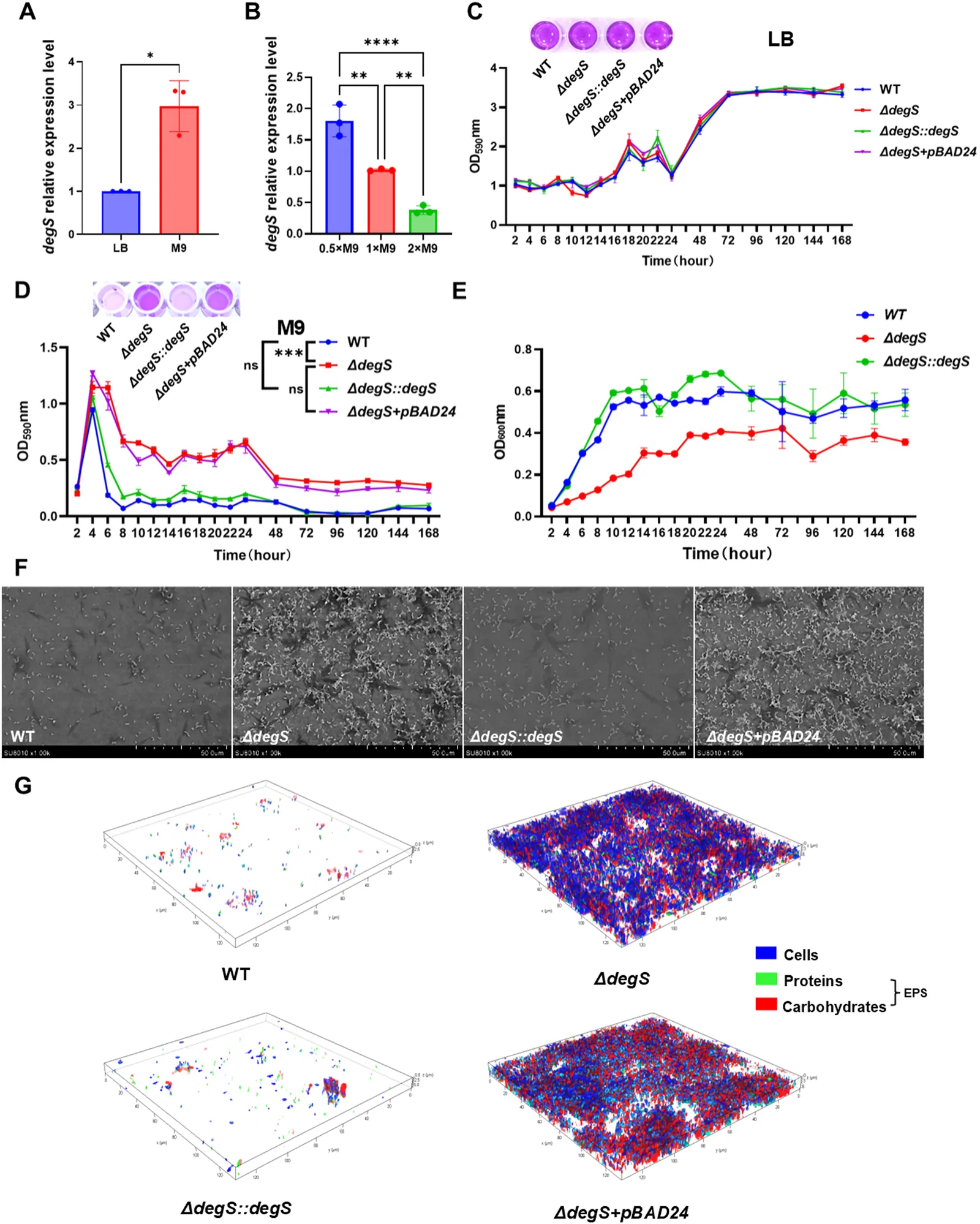
***degS* knockout enhances biofilm formation in *V. cholerae* under nutrient limiting conditions.** (A) Expression levels of *degS* in the wild-type (WT) strain cultured in LB and M9 media. (B) Expression levels of *degS* in the WT strain cultured in serially diluted M9 media (0.5×, 1×, and 2×). (A-B) The corresponding statistical values are provided in Supplementary Table 3. (C) Biofilms cultured in LB medium for 24 h (top panel: representative crystal violet (CV) staining) and 7 days (bottom panel: quantitative analysis). (D) Biofilms cultured in M9 minimal medium for 24 h (top panel: representative CV staining) and 7 days (bottom panel: quantitative analysis). (E) Growth curves of the WT, *ΔdegS*, and complemented strains in M9 medium. (F) SEM images of biofilms formed in M9 medium. (G) 3D projection images of biofilms in M9 medium acquired by CLSM. All analyses in (C–G) were performed using the WT, *ΔdegS* mutant, complemented strain (*ΔdegS::degS*), and empty vector control (*ΔdegS* + pBAD24). Student's t-tests and two-way ANOVA were used for statistical analysis, and data are presented as mean ± standard deviation (SD). *P < 0.05, **P < 0.01, ***P < 0.001, ****P < 0.0001; ns indicates no significant difference. (For interpretation of the references to colour in this figure legend, the reader is referred to the Web version of this article.)

To elucidate the function of DegS in *V. cholerae* environmental adaptation, we first determined whether its expression responds to nutritional status. qRT-PCR analysis revealed that *degS* transcription was significantly upregulated in the wild-type (WT) strain under nutrient-poor (M9) versus nutrient-rich (LB) conditions (*P* < 0.05, Fig. 1A), establishing it as a nutrient-responsive gene. To further validate that the upregulation of *degS* is specifically responsive to nutrient concentration rather than to compositional differences between LB and M9, we examined *degS* transcript levels in the WT strain cultured in serially diluted M9 media (0.5×, 1×, and 2×). As shown in Fig. 1B, *degS* expression progressively decreased as the nutrient concentration increased, with the highest expression observed in 0.5× M9 and the lowest in 2× M9. This concentration-dependent regulation confirms that DegS is a genuine nutrient-responsive regulator. In this study, nutrient limitation refers to an overall reduction in nutrient availability, as achieved by diluting all components of the M9 medium proportionally, rather than limitation of a single specific nutrient such as carbon or nitrogen alone. We hypothesized that this specific induction might regulate a key adaptive phenotype, such as biofilm formation, under low-nutrient stress. Quantification of biofilm biomass by crystal violet staining showed no difference between WT and a *ΔdegS* mutant in LB medium over 168 h (Fig. 1C). Strikingly, in M9 minimal medium, the *ΔdegS* mutant exhibited a significant and sustained enhancement in biofilm formation compared to the WT (Fig. 1D). This phenotype was fully restored to WT levels in a genetically complemented strain (*ΔdegS::degS*), confirming the specific effect of *degS* deletion. To determine whether the observed biofilm phenotype differences in M9 medium stemmed from growth phase shifts, kinetic analysis was performed for each strain. The results indicated that although the wild-type (WT), *ΔdegS* mutant, and complemented strain (*ΔdegS::degS*) exhibited different growth rates under low-nutrient conditions, all three entered the stationary phase at approximately 20 h (Fig. 1E). This demonstrates that during the key observation windows for biofilm formation (e.g. 24 h and the extended 7-day culture), each strain had already reached a comparable growth stage. Consequently, the enhanced biofilm formation observed was not due to differences in growth phase but rather resulted from specific regulatory alterations caused by the deletion of *degS*.

Microscopic analyses were employed to define the structural basis of this phenotype. Scanning electron microscopy (SEM) revealed that while WT strain in M9 medium were predominantly dispersed, the *ΔdegS* mutant formed dense, architecturally complex aggregates (Fig. 1F). Complemented strain reverted to the dispersed WT morphology. The three-dimensional architecture and composition of biofilms were further resolved by confocal laser scanning microscopy (CLSM). Consistent with the quantitative and SEM data, WT biofilms in M9 medium were sparse with minimal extracellular polymeric substance (EPS). In contrast, *ΔdegS* mutant biofilms displayed robust cellular aggregation and a substantial increase in EPS, forming mature, structurally complex 3D communities (Fig. 1G). Complementation again restored the WT biofilm morphology and EPS level. Taken together, these results demonstrate that DegS is induced by low-nutrient signals and functions as a critical repressor of biofilm matrix production and structural development under these conditions.

### 3.2 Enhanced biofilm formation in the ΔdegS mutant promotes environmental adaptation of *V. cholerae*

**Fig. 2:**
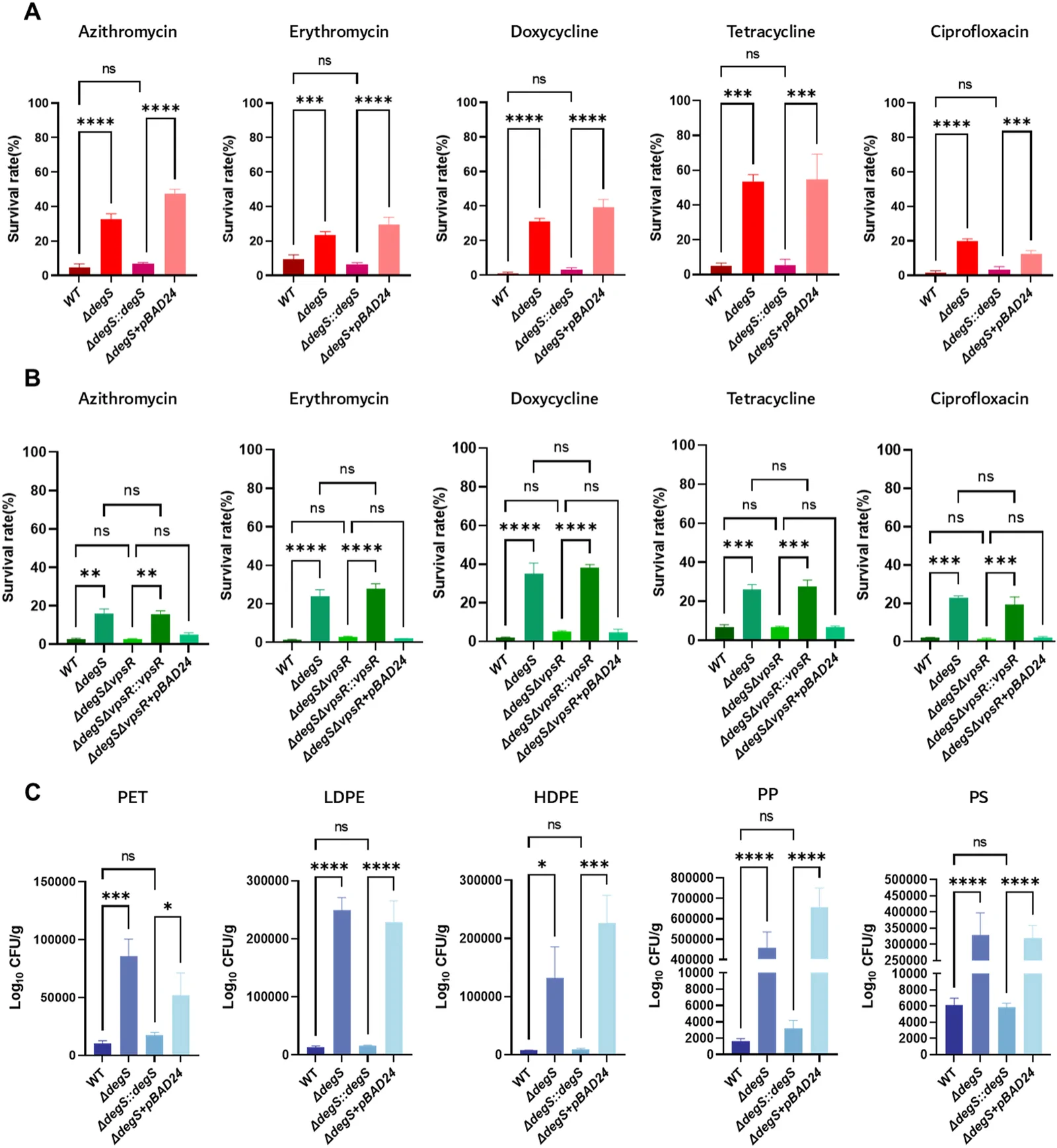
**Effects of DegS on antibiotic resistance and microplastic colonization.** (A) Survival rates of the wild-type (WT), *ΔdegS* mutant, complemented strain (*ΔdegS::degS*), and empty vector control (*ΔdegS +* pBAD24) under biofilm-forming conditions when exposed to five different antibiotics. (B) Survival rates of the WT, *ΔdegS*, *ΔdegSΔvpsR* double mutant, complemented strain (*ΔdegSΔvpsR::vpsR*), and empty vector control (*ΔdegSΔvpsR* + pBAD24) under biofilm-forming conditions when exposed to five different antibiotics. (C) Bacterial counts in biofilms formed by the indicated strains on microplastic particles (MPs) surfaces. One-way ANOVA was used for statistical analysis, and data are presented as mean ± standard deviation (SD). *P < 0.05, **P < 0.01, ***P < 0.001, ****P < 0.0001; ns indicates no significant difference.

To investigate the physiological and ecological relevance of the enhanced biofilm formation observed in the *ΔdegS* mutant, we examined its impact on antibiotic tolerance and environmental colonization. Mature biofilms grown in M9 medium were treated for 6 h with sublethal concentrations of five antibiotics commonly used against cholera—azithromycin, erythromycin, doxycycline, tetracycline, and ciprofloxacin. Under these conditions, the *ΔdegS* mutant showed a significantly higher survival rate compared to the wild type across all treatments (*P* < 0.001, Fig. 2A). This phenotype was fully reverted to wild-type levels in the genetically complemented strain (*ΔdegS::degS*), confirming that *degS* deletion directly enhances antibiotic tolerance in biofilms.

To distinguish whether this enhanced antibiotic tolerance is attributable to the hyper-biofilm phenotype itself or involves a parallel process downstream of DegS, we examined the *ΔdegSΔvpsR* double mutant, in which biofilm formation is abrogated. The *ΔdegSΔvpsR* double mutant exhibited survival rates indistinguishable from the wild type across all five antibiotics tested (Fig. 2B). Complementation with *vpsR* restored antibiotic tolerance to the *ΔdegS* level, while the empty vector control (*ΔdegSΔvpsR* + pBAD24) exhibited no restorative effect, excluding plasmid-related artifacts. These results demonstrate that the enhanced antibiotic tolerance of the *ΔdegS* mutant is strictly dependent on biofilm formation, rather than activation of a parallel resistance pathway.

Given that microplastics (MPs) have become a significant interface for microbial colonization in aquatic environments, we further assessed whether DegS influences the colonization of *V. cholerae* on MP surfaces. In co-culture experiments with five prevalent MP types—PET, LDPE, HDPE, PP, and PS—the *ΔdegS* strain colonized all tested surfaces at significantly higher levels than the wild type (*P* < 0.05). The increase was most dramatic on PP, where colonization capacity rose by 271-fold (Fig. 2C). This enhanced attachment was also *degS*-dependent, as it was abolished in the complemented strain. Collectively, these findings demonstrate that under low-nutrient conditions, loss of DegS enhances biofilm formation, which in turn not only strengthens antibiotic tolerance but also markedly promotes colonization on diverse microplastic surfaces. This underscores the role of DegS as a key negative regulator that constrains the environmental fitness and persistence of *V. cholerae*.

### 3.3 The enhanced biofilm formation in the ΔdegS mutant does not require the rpoE pathway

**Fig. 3:**
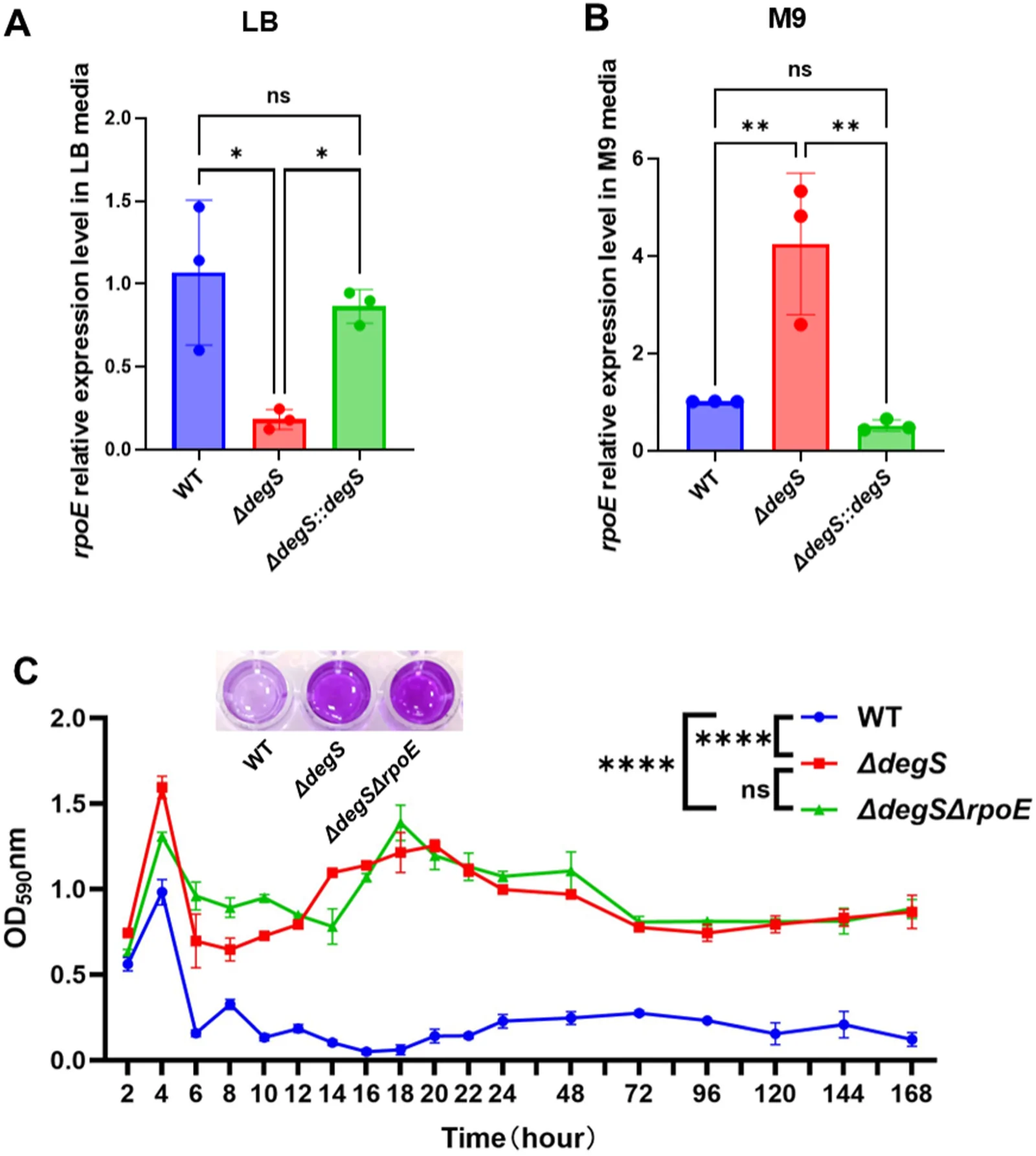
**DegS regulates *V. cholerae* biofilm formation independently of σ^E^.** Transcript levels of *rpoE* in the wild-type (WT), *ΔdegS* mutant, and complemented strain (*ΔdegS::degS*) cultured in LB (A) or M9 (B) medium. Statistical summary of the qRT-PCR data in (A) and (B) (mean ± SD, n = 3) is provided in Supplementary Table 3. (C) Biofilm formation by the WT, *ΔdegS*, and *ΔdegSΔrpoE* strains in M9 medium. Top panel: representative CV staining after 24 h of culture; bottom panel: quantitative analysis after 7 days of culture. One-way and two-way ANOVA were used for statistical analysis, and data are presented as mean ± standard deviation (SD). *P < 0.05, **P < 0.01, ***P < 0.001, ****P < 0.0001; ns indicates no significant difference.

To elucidate the molecular mechanism by which DegS inhibits biofilm formation, we first investigated whether it functions through the known σ^E^ (RpoE) stress response pathway [32]. Consistent with its canonical role as an activator of the σ^E^ pathway under nutrient-rich (LB) conditions, the transcript level of *rpoE* was significantly reduced in the *ΔdegS* mutant (Fig. 3A). However, under low-nutrient (M9) conditions, we observed an opposite trend: the *rpoE* transcript level in the *ΔdegS* mutant was dramatically up-regulated instead, and this altered expression was restored upon complementation with *degS* (Fig. 3B). This expression pattern, contrary to classical understanding, suggests that the low-nutrient environment may alter the regulatory relationship between DegS and the σ^E^ pathway.

Does this atypical activation of the σ^E^ pathway mediate the biofilm phenotype? Key phenotypic experiments demonstrated that the biofilm-forming capacity of the *ΔdegSΔrpoE* double mutant was not significantly different from that of the *ΔdegS* single mutant, and both were significantly higher than that of the wild type (Fig. 3C). This result definitively confirms that the inhibitory effect of DegS on biofilm formation under low-nutrient conditions is independent of the σ^E^ signalling pathway, indicating the existence of a novel regulatory axis.

### 3.4 The regulation of biofilm formation by DegS under nutrient-poor conditions is dependent on VpsR

**Fig. 4:**
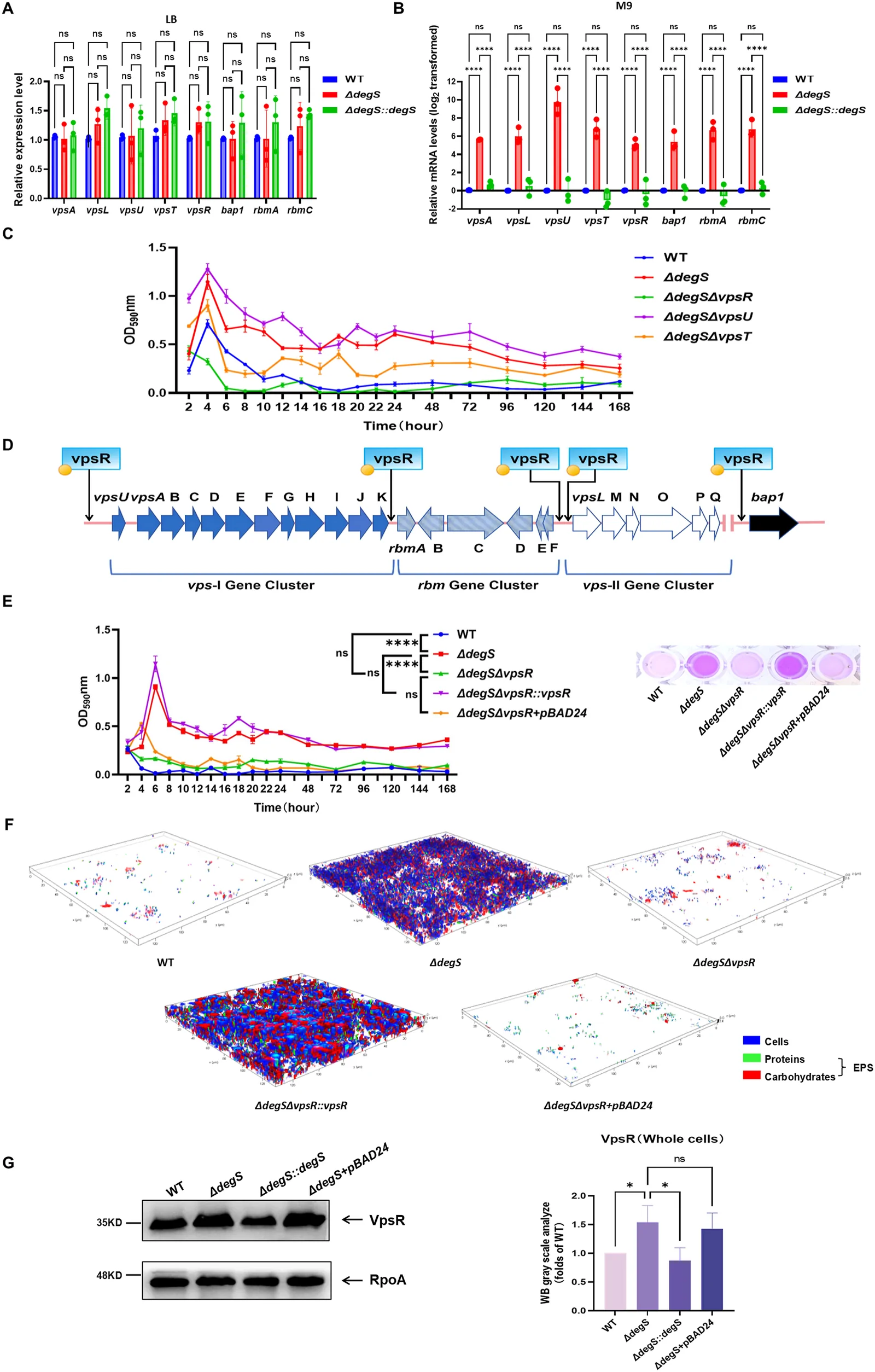
**DegS Regulates Biofilm Formation by Suppressing VpsR Protein Accumulation.** Transcript levels of biofilm-related genes in the wild-type (WT), *ΔdegS* mutant, and complemented strain (*ΔdegS*:*degS*) cultured in LB medium (A) or M9 minimal medium (B). Statistical summary of the qRT-PCR data in (A) and (B) is provided in Supplementary Table 3. Data in (B) are presented on a log_2_ scale. (C) Crystal violet (CV) staining of biofilms formed in M9 medium by the *ΔdegS* mutant and its derivative strains carrying additional deletions of *vpsR*, *vpsU*, or *vpsT*. (D) Schematic model depicting VpsR as a key transcriptional regulator that directly activates the transcription of multiple extracellular matrix synthesis genes (including the *vps-I*, *vps-II*, and *rbm* gene clusters, as well as *bap1*) by binding to the promoter regions of the exopolysaccharide (*vps*) gene clusters [33]. Discontinuous loci on the chromosome are indicated by “||”; arrows show the transcriptional direction of the genes. (E, F) Biofilm formation in M9 medium by the WT, *ΔdegS* single mutant, *ΔdegSΔvpsR* double mutant, complemented strain (*ΔdegSΔvpsR::vpsR*), and empty-vector control (*ΔdegSΔvpsR* + pBAD24). (E) Left panel: quantitative analysis of biofilm formation over a 7-day period; right panel: representative CV staining after 24 h of incubation. (F) Representative three-dimensional projection images of the biofilms. (G) Western blot analysis of VpsR protein levels in the WT, *ΔdegS* mutant, complemented strain (*ΔdegS*:*degS*), and empty-vector control (*ΔdegS* + pBAD24). RpoA was used as a loading control. Statistical significance was determined by two-tailed Student's t-test, one-way ANOVA, or two-way ANOVA as appropriate. Data are presented as mean ± SD. *P < 0.05, **P < 0.01, ***P < 0.001, ****P < 0.0001; ns, not significant. (For interpretation of the references to colour in this figure legend, the reader is referred to the Web version of this article.)

We elucidated the molecular mechanism by which DegS regulates biofilm formation under low-nutrient conditions through transcriptional analysis of eight key biofilm matrix synthesis-related genes (*vpsA*, *vpsL*, *vpsU*, *vpsT*, *vpsR*, *bap1*, *rbmA*, and *rbmC*). qRT-PCR results revealed no significant differences in the expression of these genes between the wild-type (WT) and *ΔdegS* strains when cultured in LB medium. In contrast, under M9 medium conditions, the expression of all tested genes was significantly upregulated in the *ΔdegS* mutant. This upregulation was effectively reversed upon complementation with *degS* (Fig. 4A and B), suggesting that DegS restricts the accumulation of biofilm-related gene transcripts in a low-nutrient environment.

Given that VpsR and VpsT are major regulators of biofilm formation [12], and considering the particularly pronounced upregulation of *vpsU* in the *ΔdegS* background, we further constructed *ΔdegSΔvpsR*, *ΔdegSΔvpsU*, and *ΔdegSΔvpsT* double mutants. Biofilm formation assays demonstrated that only the *ΔdegSΔvpsR* double mutant exhibited biofilm restoration to the wild-type level. Deletion of *vpsU* or *vpsT* did not reverse the enhanced phenotype of the *ΔdegS* mutant (Fig. 4C). VpsR, as a key transcriptional regulator of biofilm formation in *V. cholerae* [33], directly binds to and activates the transcription of multiple extracellular matrix synthesis-related genes, including the *Vibrio* polysaccharide (*vps*) gene clusters, the rugosity and biofilm modulation (*rbm*) gene clusters, and *bap1* (Fig. 4D) [34]. These results suggest that VpsR occupies a central position in the DegS-mediated biofilm inhibition pathway, and that DegS likely impedes downstream biofilm gene expression and matrix synthesis by suppressing VpsR.

To directly validate the functional necessity of VpsR, we constructed the *ΔdegSΔvpsR* double mutant and its complemented strain. Phenotypic analysis showed that the biofilm-forming capacity of the *ΔdegSΔvpsR* double mutant was restored to the wild-type level. Complementation with the *vpsR* gene (*ΔdegSΔvpsR::vpsR*) reinstated strong biofilm formation comparable to the *ΔdegS* single mutant, while the empty vector control (*ΔdegSΔvpsR +* pBAD24) exhibited no restorative effect (Fig. 4E). Three-dimensional imaging by CLSM further revealed that the *ΔdegSΔvpsR* biofilm was structurally sparse with significantly reduced extracellular matrix secretion, resembling the wild-type biofilm. Complementation with *vpsR* rebuilt a complex three-dimensional architecture similar to that of the *ΔdegS* mutant (Fig. 4F).

We further examined VpsR protein levels in the WT and *ΔdegS* strains by Western blot. The results demonstrated significantly elevated VpsR protein levels in the *ΔdegS* mutant, which were restored to wild-type levels in the genetically complemented strain (*ΔdegS::degS*) (Fig. 4G). Collectively, these findings establish VpsR as the central downstream effector mediating DegS-dependent regulation of biofilm formation in low-nutrient environments.

### 3.5 DegS regulates biofilm formation in *V. cholerae* under nutrient-poor conditions through the CdgH–c-di-GMP–VpsR pathway

**Fig. 5:**
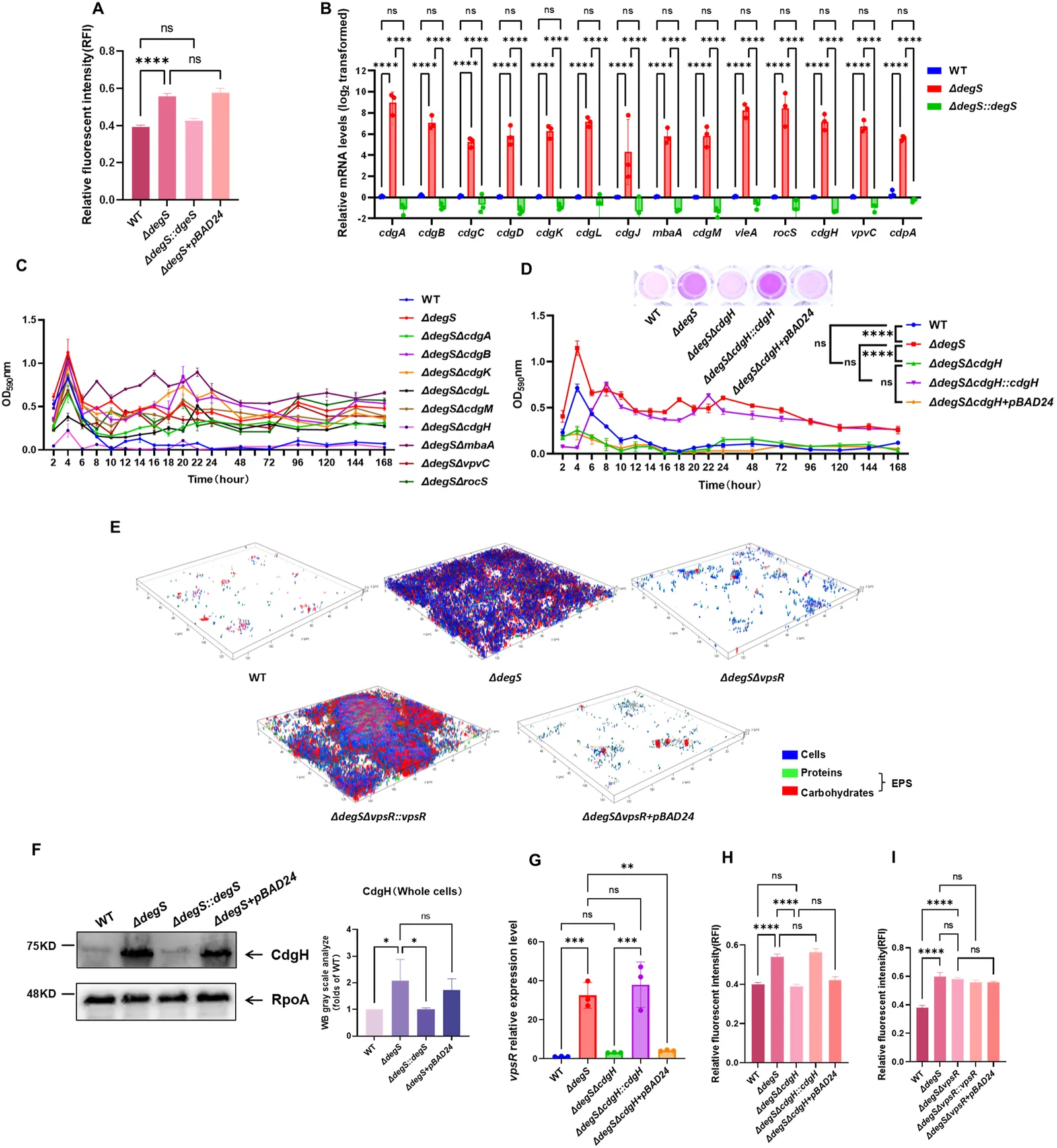
**DegS regulates biofilm formation through the CdgH-c-di-GMP-VpsR pathway.** (A) Intracellular c-di-GMP levels in biofilms of the wild-type (WT), *ΔdegS* mutant, complemented strain (*ΔdegS::degS*), and empty vector control (*ΔdegS +* pBAD24) cultured in M9 medium, measured using the Bc3-5 c-di-GMP biosensor. Relative fluorescence intensity (RFI) is expressed as the TurboRFP/AmCyan ratio (normalized internal control). (B) Transcript levels of c-di-GMP-related regulatory genes in the WT, *ΔdegS*, and complemented strain (*ΔdegS::degS*) cultured in M9 medium. The statistical summary for these genes is provided in Supplementary Table 3. Data are presented on a log_2_ scale. (C) Effect of deleting *cdgA*, *cdgB*, *cdgK, cdgL*, *cdgM*, *cdgH*, *mbaA*, *vpvC*, or *rocS* in the *ΔdegS* background on biofilm formation (crystal violet staining). (D, E) Complementation analysis of the *ΔdegSΔcdgH* double mutant in M9 medium. (D) Top panel: representative crystal violet staining after 24 h of incubation; bottom panel: quantitative analysis of biofilm formation over 7 days. (E) Representative three-dimensional projection images of biofilms. (F) CdgH protein levels detected by Western blot in the WT, *ΔdegS*, complemented strain (*ΔdegS::degS*), and empty vector control (*ΔdegS +* pBAD24), with RpoA as the loading control. (G) Transcript levels of *vpsR* in the WT, *ΔdegS*, *ΔdegSΔcdgH*, *ΔdegSΔcdgH::cdgH*, and *ΔdegSΔcdgH +* pBAD24 strains cultured in M9 medium. Statistical summary of the qRT-PCR data (mean ± SD, n = 3) is provided in Supplementary Table 3. (H) Intracellular c-di-GMP levels in the above strains indicated in panel cultured in M9 medium. (I) Intracellular c-di-GMP levels in the WT, *ΔdegS*, *ΔdegSΔvpsR*, *ΔdegSΔvpsR::vpsR*, and *ΔdegSΔvpsR +* pBAD24 strains cultured in M9 medium. One-way and two-way ANOVA were used for statistical analysis, and data are presented as mean ± standard deviation (SD). *P < 0.05, **P < 0.01, ***P < 0.001, ****P < 0.0001; ns indicates no significant difference. (For interpretation of the references to colour in this figure legend, the reader is referred to the Web version of this article.)

VpsR, a transcriptional regulator dependent on cyclic diguanosine monophosphate (c-di-GMP), binds c-di-GMP and activates extracellular matrix gene expression, thereby promoting biofilm formation [[35], [36], [37]]. To determine whether DegS influences VpsR and biofilm formation via the c-di-GMP signalling pathway, we first measured intracellular c-di-GMP levels. Using the Bc3-5 biosensor [28], we observed a highly significant increase in c-di-GMP levels in the *ΔdegS* mutant compared to the wild-type strain when cultured in M9 medium (*P* < 0.0001). This effect was reversed upon genetic complementation with *degS* (Fig. 5A), indicating that DegS negatively regulates intracellular c-di-GMP accumulation.

C-di-GMP levels are controlled by the opposing activities of diguanylate cyclases (DGCs) and phosphodiesterases (PDEs) [38]. We therefore examined the transcript levels of 13 previously reported biofilm-associated c-di-GMP metabolic enzyme genes [39]. Notably, under nutrient-poor conditions, all tested genes showed upregulation in the *ΔdegS* background (Fig. 5B). To identify the key DGCs or PDEs responsible for this phenotype, we constructed secondary deletion mutants for nine candidate positive-regulatory genes in the *ΔdegS* background. Biofilm phenotypic screening revealed that only the *ΔdegSΔcdgH* double mutant completely restored biofilm formation to wild-type level (Fig. 5C), suggesting that CdgH is the core DGC acting downstream of DegS. Genetic complementation experiments further supported this conclusion: while the *ΔdegSΔcdgH* strain exhibited wild-type biofilm levels, reintroduction of *cdgH* into this double mutant restored the enhanced biofilm phenotype seen in *ΔdegS* (Fig. 5D). CLSM three-dimensional imaging showed that the biofilm architecture and matrix secretion of *ΔdegSΔcdgH* resembled the wild-type, whereas complementation with *cdgH* re-established the complex three-dimensional structure characteristic of *ΔdegS* (Fig. 5E). Consistent with these observations, Western blot analysis confirmed that CdgH protein levels were significantly elevated in the *ΔdegS* mutant (Fig. 5F). Together, these results demonstrate that DegS inhibits biofilm formation by downregulating CdgH.

We next investigated the regulatory relationship between CdgH and VpsR. In the *ΔdegSΔcdgH* double mutant, *vpsR* transcript levels returned to wild-type levels, while complementation with *cdgH* caused its re-upregulation (Fig. 5G). Concurrent, measurements of c-di-GMP levels showed that the *ΔdegSΔcdgH* mutant exhibited c-di-GMP concentrations comparable to the wild-type, whereas levels remained high in the *ΔdegSΔvpsR* mutant (Fig. 5H and I). This confirms that CdgH acts upstream of VpsR, activating it by elevating intracellular c-di-GMP. Collectively, these findings establish that under low-nutrient conditions, DegS suppresses biofilm matrix synthesis by inhibiting CdgH, which reduces c-di-GMP levels and consequently represses the VpsR-dependent transcriptional program.

### 3.6 The DegS–CdgH–c-di-GMP–VpsR regulatory pathway is conserved and functional in natural aquatic environments

**Fig. 6:**
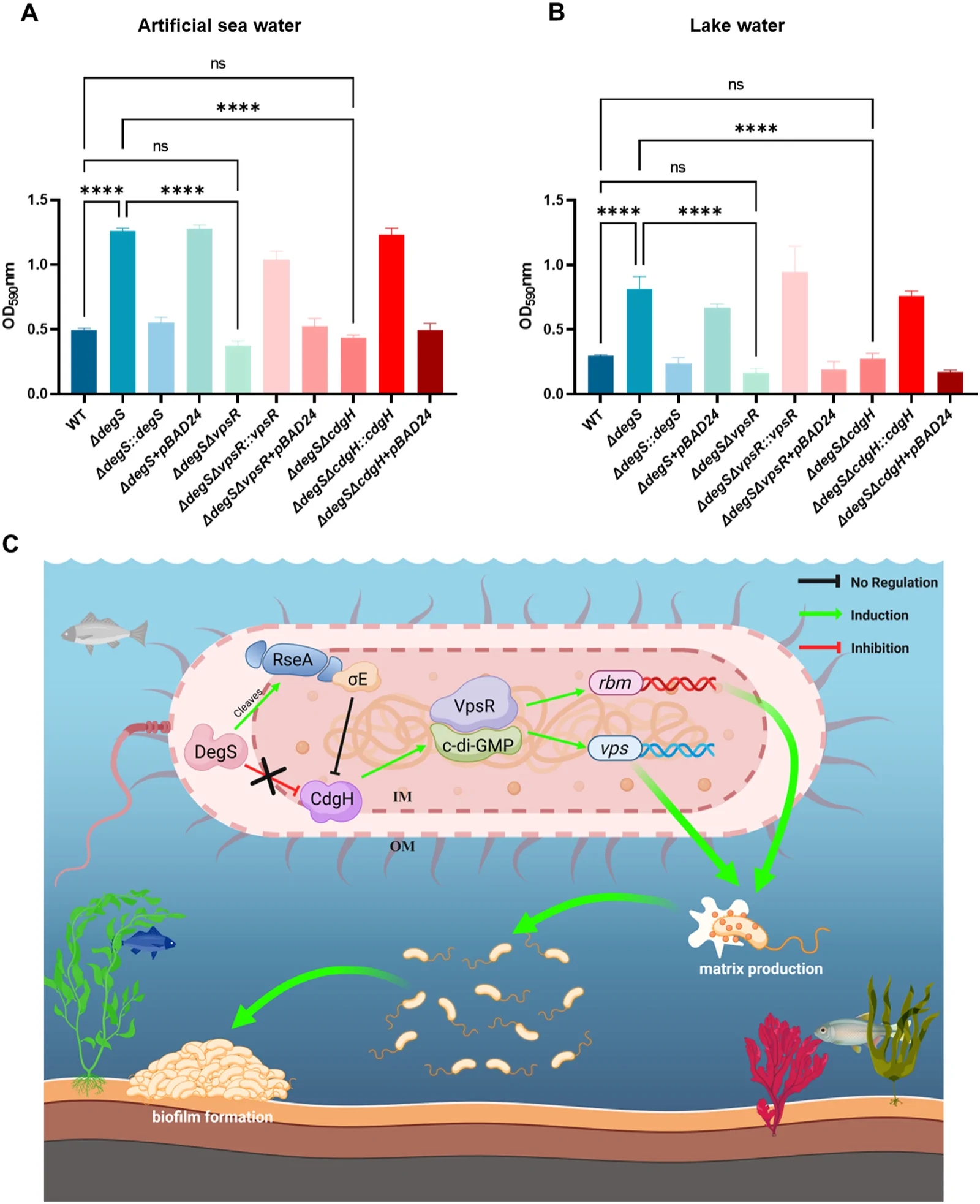
**The DegS-CdgH-c-di-GMP-VpsR pathway inhibits biofilm formation under simulated natural aquatic environments.** Quantitative evaluation of biofilm formation in relevant mutant strains under simulated aquatic environments: artificial seawater (A) and natural lake water (B). Data are presented as mean ± SD. Statistical significance was determined using two-tailed Student's *t*-tests (*P < 0.05, **P < 0.01, ***P < 0.001, ****P < 0.0001; ns, not significant). (C) Proposed model illustrating how loss of DegS enhances biofilm formation via the CdgH-c-di-GMP-VpsR axis. In the model, black T-bars denote absence of regulation, green arrows indicate activation, and red T-bars represent inhibition. (For interpretation of the references to colour in this figure legend, the reader is referred to the Web version of this article.)

To validate the biological relevance of the CdgH–c-di-GMP–VpsR pathway under environmentally realistic conditions, we recapitulated the natural habitat of *V. cholerae* using both artificial seawater and natural lake water. Quantitative biofilm analysis showed that the *ΔdegS* mutant formed significantly more biofilm than the wild-type strain in both aqueous systems (*P* < 0.05) (Fig. 6A and B), confirming that the inhibitory role of DegS is maintained across distinct water types.

Further genetic dissection revealed that in the *ΔdegS* background, deletion of either *vpsR* or *cdgH* fully abolished the hyper-biofilm phenotype. Genetic complementation with the respective gene restored biofilm formation to the *ΔdegS* level, whereas empty vector controls had no effect (Fig. 6A and B). These results were consistently reproduced in both seawater and lake water assays. Together, these findings demonstrate that the regulatory axis through which DegS suppresses biofilm formation—via CdgH, c-di-GMP, and VpsR—remains functional in simulated natural aquatic environments. This supports the conclusion that this pathway plays a conserved role in the environmental adaptation and survival of *V. cholerae*.

## 4 Discussion

DegS is a conserved periplasmic serine protease, best known as the primary activator of the σ^E^ envelope stress response pathway [40]. When outer membrane protein (OMP) misfolding occurs, DegS cleaves the anti-σ factor RseA, thereby initiating a proteolytic cascade that restores envelope homeostasis [41]. In the life cycle of *V. cholerae*, the transition from the nutrient-rich host intestine to an oligotrophic aquatic environment represents a profound physiological shock. This nutritional downshift is likely accompanied by significant perturbations in the outer membrane, which may lead to the accumulation of misfolded OMPs in the periplasm—a classic trigger for DegS activation [42]. Consistent with its role in this adaptive response, we found that *degS* expression is markedly upregulated under low-nutrient conditions (Fig. 1A). The concentration-dependent upregulation of *degS* in serially diluted M9 media (Fig. 1B) further supports that DegS responds specifically to nutrient availability rather than to compositional differences between media.

Under this specific stress, however, the conventional role of DegS appears to be fundamentally rewired. Strikingly, our genetic evidence shows that DegS represses biofilm formation independently of its canonical effector σ^E^ (RpoE). The *ΔdegSΔrpoE* double mutant exhibited a hyper-biofilm phenotype indistinguishable from that of the *ΔdegS* single mutant (Fig. 3C). This uncoupling of DegS from the σ^E^ pathway reveals a pronounced context-dependent diversification of it signalling output. Although alternative, DegS-independent mechanisms for σ^E^ activation have been reported in other bacteria under specific conditions [43], our work identifies a novel regulatory branch that originates from DegS itself during low-nutrient stress.

This branch was delineated as a precise molecular pathway: DegS-CdgH-c-di-GMP-VpsR. We demonstrate that DegS serves as a critical negative regulator of the diguanylate cyclase CdgH. Accordingly, the *ΔdegS* mutant exhibits elevated CdgH protein levels and a consequent surge in intracellular c-di-GMP (Fig. 5A–F). This increased c-di-GMP pool enhances activation of the master transcriptional regulator VpsR, leading to upregulation of biofilm matrix genes and ultimately robust biofilm development (Fig. 4, Fig. 5). The core finding that DegS inhibits biofilm formation by suppressing the CdgH-VpsR axis was rigorously validated in simulated natural aquatic environments (Fig. 6), underscoring the ecological relevance of this pathway. Notably, CdgH has been previously shown to function as a physiologically relevant DGC even in the WT background, contributing to c-di-GMP production, motility suppression, and biofilm formation [44]. Our findings extend these observations by demonstrating that DegS acts as an additional layer of negative regulation, restraining CdgH activity under low-nutrient conditions.

The physiological and ecological implications of this regulatory circuit are noteworthy. Biofilm formation represents a double-edged sword in oligotrophic environments. While it confers resistance and facilitates surface colonization, excessive or premature commitment to a sessile lifestyle could trap a population in a suboptimal niche, thereby limiting dispersal and the search for new resources [45]. By acting as a brake on the CdgH-VpsR-mediated biofilm program, DegS likely fine-tunes this critical lifestyle decision. Its upregulation under nutrient scarcity may temper biofilm development, thereby maintaining a reservoir of motile or loosely attached cells capable of dispersing to locate more favorable microenvironments [46]. This strategic flexibility aligns with broader paradigms in microbial ecology, in which regulatory networks optimize the trade-off between attachment and dispersal in response to environmental cues [47].

Conversely, loss of DegS function locks the population into a hyper-biofilm state, as demonstrated in our experiments. This state enhances short-term survival traits, including significantly increased antibiotic tolerance within biofilms and a dramatically elevated capacity to colonize environmentally relevant surfaces such as microplastics [48]. In an era of ubiquitous microplastic pollution, understanding the regulatory pathways that govern pathogen attachment to these particles is crucial for assessing environmental reservoirs and transmission risks [27].

A key mechanistic question emerging from this work concerns the nature of the link between the periplasmic protease DegS and the inner membrane-associated diguanylate cyclase CdgH. Our current data do not clarify the direct molecular steps underlying this regulation interaction. It remains unknown whether DegS proteolytically processes an intermediate signalling component that modulates CdgH activity, or whether the connection is more indirect, involving broader periplasmic or cellular state changes sensed by CdgH. Furthermore, the precise nature of the nutrient-derived signal that triggers DegS activation-whether it involves outer membrane protein misfolding, changes in periplasmic osmolarity, or another cue-warrants further investigation. While we observed that *degS* expression is upregulated under low-nutrient conditions (Fig. 1A), the molecular details of how DegS senses nutrient limitation and transduces this signal specifically to CdgH remain to be elucidated. Elucidating this “missing link” represents an important direction for future research. Furthermore, integrating this newly identified DegS-dependent pathway with other well-characterised networks that sense nutrient limitation (e.g. PhoBR for phosphate [10,49], Fur/RyhB for iron [11,50]) and regulate c-di-GMP will be essential for building a holistic model of how *V. cholerae* coordinates its survival strategy in the environment.

In summary, this study expands the functional scope of DegS in *Vibrio cholerae*. We propose a model in which periplasmic stress induced by nutrient limitation—likely mediated through the accumulation of misfolded outer membrane proteins—activates DegS. Rather than channeling its signal exclusively through the σ^E^ pathway for envelope repair, DegS simultaneously and independently engages a novel regulatory axis that suppresses biofilm formation via the CdgH–c-di-GMP–VpsR cascade (Fig. 6C). This pathway acts as an environmentally tuned rheostat, modulating the shift to a sessile lifestyle and thereby influencing key phenotypes such as antibiotic resilience and colonization of aquatic interfaces, including microplastics. By connecting a central periplasmic sentinel to the core regulatory hub of biofilm development, our findings offer new molecular insight into the adaptive strategies that support the environmental persistence and dissemination of a major human pathogen. Moreover, this work highlights DegS as a potential target for intervention strategies aimed at disrupting the environmental reservoir of cholera.

## CRediT authorship contribution statement

**Xiaoyu Huang:** Writing – original draft, Methodology, Formal analysis, Data curation. **Tao Chen:** Writing – original draft, Methodology, Data curation. **Qiqi Linghu:** Writing – original draft, Methodology, Formal analysis. **Hui Wang:** Investigation, Funding acquisition, Formal analysis. **Shilu Luo:** Methodology. **Huan Zhou:** Methodology. **Xun Min:** Writing – review & editing, Project administration, Funding acquisition, Data curation, Conceptualization. **Jian Huang:** Writing – review & editing, Project administration, Funding acquisition, Data curation, Conceptualization.

## Funding

This work was supported by grants from the 10.13039/501100001809National Natural Science Foundation of China (No. 82360397), the Research and Talent Training Project of Guizhou Moutai Hospital (MTyk 2022-08), Zunyi Science and Technology Program Project (Zun Shi Ke He HZ (2023) 261).

## Declaration of competing interest

The authors declare that they have no known competing financial interests or personal relationships that could have appeared to influence the work reported in this paper.

## Data Availability

Data will be made available on request.
