# Supplementary material for: A nutrient-sensing protease DegS suppresses biofilm formation in Vibrio cholerae via the CdgH–c-di-GMP–VpsR axis under nutrient limitation
Source: Biofilm. 2026 Sep 10;12:100398. doi: 10.1016/j.bioflm.2026.100398 (PMC13594642; doi:10.1016/j.bioflm.2026.100398)
Supplement: Supplementary file 1 [file mmc1.docx]

***Supplementary Material***

**Supplementary Table 1 The information of bacterial strains and plasmids used in this study**

| **Strains or plasmids** | **Genotype or feature(s)** | **Reference or source** |
| --- | --- | --- |
| **Strains** |  |  |
| Non-O1/non-O139 *Vibrio cholerae* Strains |  |  |
| HN375 | Wild-type non-O1/non-O139 *V. cholerae* | Lab collections |
| *ΔdegS* | in-frame deletion of *degS* | Lab collections |
| *ΔdegS::degS* | *ΔdegS* complemented with pBAD24-*degS*, Amp^r^ | Lab collections |
| *ΔdegS*+pBAD24 | *ΔdegS* complemented with pBAD24, Amp^r^ | Lab collections |
| *ΔdegSΔrpoE* | in-frame deletion of *degS* and *rpoE* | Lab collections |
| *ΔdegSΔvpsU* | in-frame deletion of *degS* and *vpsU* | This study |
| *ΔdegSΔvpsT* | in-frame deletion of *degS* and *vpsT* | This study |
| *ΔdegSΔvpsR* | in-frame deletion of *degS* and *vpsR* | This study |
| *ΔdegSΔvpsR::vpsR* | *ΔdegSΔvpsR* complemented with pBAD24-*vpsR*, Amp^r^ | This study |
| *ΔdegSΔvpsR*+pBAD24 | *ΔdegSΔvpsR* complemented with pBAD24, Amp^r^ | This study |
| *ΔdegSΔcdgA* | in-frame deletion of *degS* and *cdgA* | This study |
| *ΔdegSΔcdgB* | in-frame deletion of *degS* and *cdgB* | This study |
| *ΔdegSΔcdgK* | in-frame deletion of *degS* and *cdgK* | This study |
| *ΔdegSΔcdgL* | in-frame deletion of *degS* and *cdgL* | This study |
| *ΔdegSΔcdgM* | in-frame deletion of *degS* and *cdgM* | This study |
| *ΔdegSΔcdgH* | in-frame deletion of *degS* and *cdgH* | This study |
| *ΔdegSΔmbaA* | in-frame deletion of *degS* and *mbaA* | This study |
| *ΔdegSΔvpvC* | in-frame deletion of *degS* and *vpvC* | This study |
| *ΔdegSΔrocS* | in-frame deletion of *degS* and *rocS* | This study |
| *ΔdegSΔcdgH::cdgH* | *ΔdegSΔcdgH* complemented with pBAD24-*cdgH*, Amp^r^ | This study |
| *ΔdegSΔcdgH*+pBAD24 | *ΔdegSΔcdgH* complemented with pBAD24, Amp^r^ | This study |
| *E. coli* Strains |  |  |
| DH5α | F−, ø80dlacZ*Δ*M15, *Δ*(lacZYA-argF) U169 deoR, recA1, endA1, hsdR17 (rk-,mk+), phoA, supE44, ʎ-, thi-1, gyrA96, relA1 | Lab collections |
| DH5α( λpir) | supE44 *Δ*lacU169 (ΦlacZ*Δ*M15) recA1 endA1 hsdR17 thi-1 gyrA96 relA1 λpir | Lab collections |
| WM3064 | thrB1004 pro thi rpsL hsdS lacZ*Δ*M15 RP4-1360 *Δ*(araBAD)567 *Δ*dapA1341::[erm pir] | Lab collections |
| BL21 | F-, ompT, hsdSB (rB-mB-), gal, dcm | Lab collections |
| pCold TF-*vpsR*-BL21 | BL21 complemented with pCold TF-*vpsR*, Amp^r^ | This study |
| pCold TF-*cdgH*-BL21 | BL21 complemented with pCold TF-*cdgH*, Amp^r^ | This study |
| **Plasmids** |  |  |
| pWM91 | Suicide plasmid; oriR oriT lacZ tetAR sacB, Amp^r^ | Lab collections |
| pWM91-*ΔvpsR* | pWM91 carrying upstream and downstream fragments flanking *vpsR*, Amp^r^ | This study |
| pWM91-*ΔcdgH* | pWM91 carrying upstream and downstream fragments flanking *cdgH*, Amp^r^ | This study |
| pBAD24 | Expression vector with araBAD promoter and rrnB T1 terminator, Amp^r^ | Lab collections |
| pBAD24-*vpsR* | pBAD24 expressing VpsR, Amp^r^ | This study |
| pBAD24-*cdgH* | pBAD24 expressing CdgH, Amp^r^ | This study |
| pCold TF | Expression vector with N-His, N-Trigger factor, N-HRV 3C, N-Thrombin, N-Factor Xa tag, Amp^r^ | Lab collections |
| pCold TF-*vpsR* | pCold TF expressing VpsR | This study |
| pCold TF-*cdgH* | pCold TF expressing CdgH | This study |
| pRP0122-Pbe-amcyan_Bc3-5_turborfp | The c-di-GMP biosensor consists of AmCyan, a triple-tandem c-di-GMP-I domain, and TurboRFP, Spe^r^ | Prof. Jin He |

**Supplementary Table 2 The list of the primers used in the study**

| **Name** | **Primer sequence (forward/reverse, 5’ to 3’)** | **Use and description** |
| --- | --- | --- |
| Primers for mutant construction | | |
| *vpsU* up-F | TCCCCCGGGCTATTCCTTTGATCGTCTCAACAG | For construction of deletion mutant of *ΔdegSΔvpsU* |
| *vpsU* up-R | CGCATTTATCCCCAGCTCTTGCCTGTACACACGACTAATACTG |  |
| *vpsU* down-F | CAGTATTAGTCGTGTGTACAGGCAAGAGCTGGGGATAAATGCG |  |
| *vpsU* down-R | CCGCTCGAGGCTAAAGAGGCAGCAAACGTGGTAG |  |
| *vpsT* up-F | TCCCCCGGGGTGCTAACCCCACAATTAGTC | For construction of deletion mutant of *ΔdegSΔvpsT* |
| *vpsT* up-R | CAATATTAGAAGCACCACTACCTTCACCCCTCCTAACACATCAAGG |  |
| *vpsT* down-F | CCTTGATGTGTTAGGAGGGGTGAAGGTAGTGGTGCTTCTAATATTG |  |
| *vpsT* down-R | CCGCTCGAGGATTGAGACAATAAGGGAACTTCG |  |
| *vpsR* up-F | TCCCCCGGGCAACTTTCATCACTGGCTACC | For construction of deletion mutant of *ΔdegSΔvpsR* |
| *vpsR* up-R | CAGTAAGCGATACATGGTTGCCCATACGGAATTGAGTGCTCAT |  |
| *vpsR* down-F | ATGAGCACTCAATTCCGTATGGGCAACCATGTATCGCTTACTG |  |
| *vpsR* down-R | CCGCTCGAGCGAACCAGTAACACAGCTAATTG |  |
| *cdgA* up-F | TCCCCCGGGCACAATCGATATCCACACGACT | For construction of deletion mutant of *ΔdegSΔcdgA* |
| *cdgA* up-R | AACTGGCTCAAATTGGTGCTGTCCGACATGAAGATCAGCAGTGT |  |
| *cdgA* down-F | ACACTGCTGATCTTCATGTCGGACAGCACCAATTTGAGCCAGTT |  |
| *cdgA* down-R | CCGCTCGAGGACCATGGATGGCCAAGATGTG |  |
| *cdgB* up-F | CGCGGATCCGACATGCTCATCGGTCACATT | For construction of deletion mutant of *ΔdegSΔcdgB* |
| *cdgB* up-R | GGCTATTCATGCCAAGTGGTACCGACCTGATTCCTAATGAGTG |  |
| *cdgB* down-F | TCACTCATTAGGAATCAGGTCGGTACCACTTGGCATGAATAGCC |  |
| *cdgB* down-R | TCCCCCGGGAGTTGGTGGAGTGCGATAGTC |  |
| *cdgK* up-F | TCCCCCGGGGTTCCAAGAACGGATCAGCATC | For construction of deletion mutant of *ΔdegSΔcdgK* |
| *cdgK* up-R | CTCTATCCCACCAGAGATGGTAAGTGGCGCACAGATAATCCAAG |  |
| *cdgK* down-F | CTTGGATTATCTGTGCGCCACTTACCATCTCTGGTGGGATAGAG |  |
| *cdgK* down-R | CCGCTCGAGTTAGGTTGGGAAGATCCGGAAG |  |
| *cdgL* up-F | CGCGGATCCTTCGCTTAACGCAGATCAGCAG | For construction of deletion mutant of *ΔdegSΔcdgL* |
| *cdgL* up-R | CAACGTATCGATCTCGTCCGTGCATTGAGTGTCGTTAGCCAGCG |  |
| *cdgL* down-F | CGCTGGCTAACGACACTCAATGCACGGACGAGATCGATACGTTG |  |
| *cdgL* down-R | TCCCCCGGGCCACCCTGCACAATCAAGATCG |  |
| *cdgM* up-F | TCCCCCGGGCTAGCCCAGCATGGAGCATATC | For construction of deletion mutant of *ΔdegSΔcdgM* |
| *cdgM* up-R | GTACTCACAGTTAATGCTCGCCGTACCAGCAAGGGCACAATCAC |  |
| *cdgM* down-F | GTGATTGTGCCCTTGCTGGTACGGCGAGCATTAACTGTGAGTAC |  |
| *cdgM* down-R | CCGCTCGAGTGACGCCCTTCCCATTCAATAC |  |
| *cdgH* up-F | CGCGGATCCGTGCTTATTCCGCTGTTGATGG | For construction of deletion mutant of *ΔdegSΔcdgH* |
| *cdgH* up-R | GAACAGCATTTCACCAGTTTGAGGTGGAAATCAATAGCCAACCT |  |
| *cdgH* down-F | AGGTTGGCTATTGATTTCCACCTCAAACTGGTGAAATGCTGTTC |  |
| *cdgH* down-R | TCCCCCGGGCTTCGGTAAGTTCAGCCACACA |  |
| *mbaA* up-F | CGCGGATCCCATGCGGTACCTTATTTCTG | For construction of deletion mutant of *ΔdegSΔmbaA* |
| *mbaA* up-R | AATACGGTGCTAAAGGCACTTCTTGGTAAGTCACACTGCTGATG |  |
| *mbaA* down-F | CATCAGCAGTGTGACTTACCAAGAAGTGCCTTTAGCACCGTATT |  |
| *mbaA* down-R | TCCCCCGGGGAAGATGAGAAAGCGGCATTAG |  |
| *vpvC* up-F | CGCGGATCCGGCAGAGGTACAGAAATGGT | For construction of deletion mutant of *ΔdegSΔvpvC* |
| *vpvC* up-R | CTATCTGAACTGATCCTGCTAGCAAGCGATTAACCAGTGGCG |  |
| *vpvC* down-F | CGCCACTGGTTAATCGCTTGCTAGCAGGATCAGTTCAGATAG |  |
| *vpvC* down-R | TCCCCCGGGCTTGATGGACATTCAAATGCCG |  |
| *rocS* up-F | CGCGGATCCGTAGATCCACGCTATCCATCAG | For construction of deletion mutant of *ΔdegSΔrocS* |
| *rocS* up-R | CGCAAACAACTCTTCCACTTCGGAGATGAGGTTTGAGCAGGCAT |  |
| *rocS* down-F | ATGCCTGCTCAAACCTCATCTCCGAAGTGGAAGAGTTGTTTGCG |  |
| *rocS* down-R | TCCCCCGGGCAGCTCGACACCTTGTGGAAT |  |
| Primers for constructs for complementation | | |
| *vpsR* -F | CCGGAATTCATGAGCACTCAATTCCGTATGGAT | For cloning complete length of *vpsR* into pBAD24 |
| *vpsR* -R | CCCAAGCTTTTAGAAGTTTTCATCGGTGATC |  |
| *cdgH* -F | TCCCCCGGGATGCTACTTAACGCTTTTTCAC | For cloning complete length of *cdgH* into pBAD24 |
| *cdgH* -R | TGCTCTAGATTAAACGATGTGTAATCCTTTT |  |
| Primers for constructs for expressing protein | | |
| TF-*vpsR*-F | CCGGAATTCATGAGCACTCAATTCCGTATGGAT | For cloning *vpsR* into pCold TF plasmid |
| TF-*vpsR*-R | CCCAAGCTTAGTTTTCATCGGTGATCAAGTT |  |
| TF-*cdgH*-F | CGCGGATCCATGCTACTTAACGCTTTTTCAC | For cloning *cdgH* into pCold TF plasmid |
| TF-*cdgH*-R | CCGGAATTCTTAAACGATGTGTAATCCTTTT |  |
| Primers for constructs for c-di-GMP reporter strain | | |
| *turborfp*_F | CAAAACAGATAGGAGGAATTTTCCATGGGAAGTGAATTGATTAAAGAAAATATGCATATG | For cloning pRP0122-Pbe-amcyan_Bc3-5_turborfp Plasmids into c-di-GMP reporter strain |
| *turborfp*_R | GGGGTACCACTAGTAAGCTTCTCGAGTTATCTATGCCCTAATTTACTAGG |  |
| Primers for Real time PCR | | |
| q*-16s RNA-*F | CGGTAATACGGAGGGTGCAA | For qRT-PCR to targeting 16sRNA |
| q*-16s RNA* *-*R | CACCTGCATGCGCTTTACG |  |
| q*-degS-*F | CAACTACCACGTCGTCGCGCAG | For qRT-PCR to targeting *degS* |
| q*-degS-*R | GTTACCAATCGCCAACACCACG |  |
| q*-rpoE-*F | TATCGAACCCGGAGAACATT | For qRT-PCR to targeting *rpoE* |
| q*-rpoE-*R | TTTCCTCGTAACTCAAGCCA |  |
| q*-vpsA-*F | GCTGATTCTGGTAACAGGAC | For qRT-PCR to targeting *vpsA* |
| q*-vpsA-*R | AAGGCAAATACTGCTGCGG |  |
| q*-vpsL-*F | CGCAGTATGCGAGTGATGG | For qRT-PCR to targeting *vpsL* |
| q*-vpsL-*R | CGACATACTTCCTTGCAAC |  |
| q*-vpsU-*F | CGATATCTGTCCTGCTGAGC | For qRT-PCR to targeting *vpsU* |
| q*-vpsU-*R | CATTTATCCCCAGCTCTTGCC |  |
| q*-vpsT-*F | TGTCCGCAGGATATTGAGCATAAGC | For qRT-PCR to targeting *vpsT* |
| q*-vpsT-*R | ACTCATGCCTTTGATCAGGGTATCC |  |
| q*-vpsR-*F | CTACATTAACGTATTGCGG | For qRT-PCR to targeting *vpsR* |
| q*-vpsR-*R | GCATCATCAGAGAAGCTAC |  |
| q*-bap1-*F | TCTACCAGCGTTTTTCATCCT | For qRT-PCR to targeting *bap1* |
| q*-bap1-*R | GTCAGCCACATCTTTCTCATC |  |
| q*-rbmA-*F | CCGGCGGTTTTGTTCCATTC | For qRT-PCR to targeting *rbmA* |
| q*-rbmA-*R | GAATAAGTATCACCTGAAGC |  |
| q*-rbmC-*F | CTCGGTTCGTTCAGGATTTGCG | For qRT-PCR to targeting *rbmC* |
| q*-rbmC-*R | GCGCTAATAGATGATAACCACCCC |  |
| q*-cdgA-*F | GCATACTAACCAAGCGATCTGG | For qRT-PCR to targeting *cdgA* |
| q*-cdgA-*R | GTTGTTTAAAGCGCGCAAACG |  |
| q*-cdgB-*F | CAACCGTTACGCAATGGATTCG | For qRT-PCR to targeting *cdgB* |
| q*-cdgB-*R | GCTTTGCATCGACTCTGGTCAT |  |
| q*-cdgC-*F | GGTTACCACCTTTGGGATGATG | For qRT-PCR to targeting *cdgC* |
| q*-cdgC-*R | CTTGCCCAGCGTGAATTAAACG |  |
| q*-cdgK-*F | GATCAGAGAGCAACATGAGTGG | For qRT-PCR to targeting *cdgK* |
| q*-cdgK-*R | CATATTCACTGGTGAGCTTGTG |  |
| q*-cdgL-*F | GGAGGATGAGCCCTATTTAG | For qRT-PCR to targeting *cdgL* |
| q*-cdgL-*R | GAAACATCCACTTCGTCTTGC |  |
| q*-cdgJ-*F | CTACGTCAGTCCAGAGCATGTG | For qRT-PCR to targeting *cdgJ* |
| q*-cdgJ-*R | CCTGTCCAAGATAAACCAAGGC |  |
| q*-mbaA-*F | CGATCCATTTGCCGAGCTTGAC | For qRT-PCR to targeting *mbaA* |
| q*-mbaA-*R | CTCAAGCACCACATACACCACC |  |
| q*-cdgM-*F | GAAGATGATCGCAGCTTACTTG | For qRT-PCR to targeting *cdgM* |
| q*-cdgM-*R | GATAAAGAACCGGACTGTCTTC |  |
| q*-vieA-*F | GTTACGAGCACCCAACTCATGG | For qRT-PCR to targeting *vieA* |
| q*-vieA-*R | CAAGTTGCGCTGGCTGATGTTG |  |
| q*-rocS-*F | CATATTGATAGCCTCACTGG | For qRT-PCR to targeting *rocS* |
| q*-rocS-*R | CACTTTGTCACCGATGTGATG |  |
| q*-cdgH-*F | GTTAAGCTAGAGAACAACCCGC | For qRT-PCR to targeting *cdgH* |
| q*-cdgH-*R | GCTTTCACTGAAGTAAGCGAGG |  |
| q*-vpvC-*F | CTTGGTCGGTAAACCCCTGACG | For qRT-PCR to targeting *vpvC* |
| q*-vpvC-*R | CAATCTGTTGACGCAGCTCGCG |  |
| q*-cdpA-*F | CACGATTTGCCTTACGCGACAC | For qRT-PCR to targeting *cdpA* |
| q*-cdpA-*R | CGCCAGTTTCGACATAAAGCGC |  |
