# Supplementary material for: A nutrient-sensing protease DegS suppresses biofilm formation in Vibrio cholerae via the CdgH–c-di-GMP–VpsR axis under nutrient limitation
Source: Biofilm. 2026 Sep 10;12:100398. doi: 10.1016/j.bioflm.2026.100398 (PMC13594642; doi:10.1016/j.bioflm.2026.100398)
Supplement: Supplementary file 2 [file mmc2.docx]

**Supplementary Table 3. Statistical summary of qRT‑PCR data for all genes analyzed in this study.**

**Figure 1A**

|  | **WT (LB)** | | **WT (M9)** | |
| --- | --- | --- | --- | --- |
| **Gene** | **Mean** | **SD** | **Mean** | **SD** |
| ***degS*** | 1.00 | 0.00 | 2.98 | 0.59 |

Expression levels of *degS* in the WT strain cultured in LB and M9 media. Data are presented as mean ± SD from three independent biological replicates (n=3). The relative expression of each gene was calculated using the 2^⁻ΔΔCt^ method, with 16S rRNA as the internal control and the WT strain cultured in LB medium as the calibrator (set to 1).

**Figure 1B**

|  | **WT (0.5×M9)** | | **WT (1×M9)** | | **WT (2×M9)** | |
| --- | --- | --- | --- | --- | --- | --- |
| **Gene** | **Mean** | **SD** | **Mean** | **SD** | **Mean** | **SD** |
| ***degS*** | 1.80 | 0.25 | 1.02 | 0.02 | 0.38 | 0.07 |

*degS* expression levels in the WT strain cultured in serially diluted M9 media (0.5×, 1×, and 2×). Data are presented as mean ± SD from three independent biological replicates (n=3). The relative expression of each gene was calculated using the 2^⁻ΔΔCt^ method, with 16S rRNA as the internal control and the WT strain cultured in 1× M9 as the calibrator (set to 1).

**Figure 3A**

| **LB** | **WT** | | ***ΔdegS*** | | ***ΔdegS::degS*** | |
| --- | --- | --- | --- | --- | --- | --- |
| **Gene** | **Mean** | **SD** | **Mean** | **SD** | **Mean** | **SD** |
| ***rpoE*** | 1.07 | 0.44 | 0.18 | 0.06 | 0.86 | 0.10 |

Transcript levels of *rpoE* in the WT, *ΔdegS* mutant, and complemented strain (*ΔdegS::degS*) cultured in LB medium. Data are presented as mean ± SD from three independent biological replicates (n=3). The relative expression of each gene was calculated using the 2^⁻ΔΔCt^ method, with 16S rRNA as the internal control and the WT strain as the calibrator (set to 1).

**Figure 3B**

| **M9** | **WT** | | ***ΔdegS*** | | ***ΔdegS::degS*** | |
| --- | --- | --- | --- | --- | --- | --- |
| **Gene** | **Mean** | **SD** | **Mean** | **SD** | **Mean** | **SD** |
| ***rpoE*** | 1.01 | 0.00 | 4.25 | 1.46 | 0.52 | 0.12 |

Transcript levels of *rpoE* in the WT, *ΔdegS* mutant, and complemented strain (*ΔdegS::degS*) cultured in M9 medium. Data are presented as mean ± SD from three independent biological replicates (n=3). The relative expression of each gene was calculated using the 2^⁻ΔΔCt^ method, with 16S rRNA as the internal control and the WT strain as the calibrator (set to 1).

**Figure 4A**

| **LB** | **WT** | | ***ΔdegS*** | | ***ΔdegS::degS*** | |
| --- | --- | --- | --- | --- | --- | --- |
| **Gene** | **Mean** | **SD** | **Mean** | **SD** | **Mean** | **SD** |
| ***vpsA*** | 1.05 | 0.03 | 1.02 | 0.23 | 1.08 | 0.24 |
| ***vpsL*** | 1.02 | 0.03 | 1.27 | 0.29 | 1.54 | 0.18 |
| ***vpsU*** | 1.05 | 0.04 | 1.07 | 0.48 | 1.20 | 0.40 |
| ***vpsT*** | 1.07 | 0.09 | 1.34 | 0.26 | 1.46 | 0.22 |
| ***vpsR*** | 1.03 | 0.02 | 1.31 | 0.20 | 1.32 | 0.34 |
| ***bap1*** | 1.02 | 0.01 | 1.02 | 0.30 | 1.29 | 0.54 |
| ***rbmA*** | 1.03 | 0.03 | 1.02 | 0.49 | 1.30 | 0.46 |
| ***rbmC*** | 1.03 | 0.02 | 1.24 | 0.40 | 1.44 | 0.07 |

 Transcript levels of biofilm‑related genes in the WT, *ΔdegS* mutant, and complemented strain (*ΔdegS::degS*) cultured in LB medium. Data are presented as mean ± SD from three independent biological replicates (n=3). The relative expression of each gene was calculated using the 2^⁻ΔΔCt^ method, with 16S rRNA as the internal control and the WT strain as the calibrator (set to 1).

**Figure 4B**

| **M9** | **WT** | | ***ΔdegS*** | | ***ΔdegS::degS*** | |
| --- | --- | --- | --- | --- | --- | --- |
| **Gene** | **Mean** | **SD** | **Mean** | **SD** | **Mean** | **SD** |
| ***vpsA*** | 0.06 | 0.03 | 5.63 | 0.04 | 0.68 | 0.31 |
| ***vpsL*** | 0.01 | 0.01 | 5.99 | 0.88 | 0.53 | 0.96 |
| ***vpsU*** | 0.02 | 0.01 | 9.75 | 1.44 | -0.11 | 1.26 |
| ***vpsT*** | 0.02 | 0.01 | 6.83 | 0.98 | -1.06 | 0.91 |
| ***vpsR*** | 0.03 | 0.02 | 5.11 | 0.54 | -0.37 | 1.43 |
| ***bap1*** | 0.02 | 0.01 | 5.40 | 1.01 | -0.06 | 0.69 |
| ***rbmA*** | 0.01 | 0.00 | 6.66 | 0.98 | -0.61 | 1.13 |
| ***rbmC*** | 0.03 | 0.01 | 6.79 | 0.91 | 0.31 | 0.66 |

 Transcript levels of biofilm‑related genes in the WT, *ΔdegS* mutant, and complemented strain (*ΔdegS::degS*) cultured in M9 minimal medium. Data are presented as mean ± SD from three independent biological replicates (n=3). The relative expression of each gene was calculated using the 2^⁻ΔΔCt^ method, with 16S rRNA as the internal control and the WT strain as the calibrator (set to 1). Data are presented on a log₂ scale.

**Figure 5B**

|  | **WT** | | ***ΔdegS*** | | ***ΔdegS::degS*** | |
| --- | --- | --- | --- | --- | --- | --- |
| **Gene** | **Mean** | **SD** | **Mean** | **SD** | **Mean** | **SD** |
| ***cdgA*** | 0.07 | 0.06 | 8.96 | 1.04 | -1.13 | 0.50 |
| ***cdgB*** | 0.17 | 0.08 | 7.05 | 0.63 | -0.90 | 0.28 |
| ***cdgC*** | 0.10 | 0.08 | 5.22 | 0.45 | -0.67 | 0.87 |
| ***cdgD*** | 0.05 | 0.04 | 5.83 | 0.91 | -1.30 | 0.30 |
| ***cdgK*** | 0.05 | 0.06 | 6.26 | 0.63 | -0.91 | 0.27 |
| ***cdgL*** | 0.04 | 0.04 | 7.13 | 0.44 | -0.80 | 1.22 |
| ***cdgJ*** | 0.02 | 0.01 | 4.28 | 3.08 | -1.33 | 1.33 |
| ***mbaA*** | 0.05 | 0.04 | 5.79 | 0.73 | -1.25 | 0.08 |
| ***cdgM*** | 0.01 | 0.01 | 5.81 | 0.99 | -1.43 | 0.44 |
| ***vieA*** | 0.06 | 0.05 | 8.24 | 0.65 | -0.69 | 0.45 |
| ***rocS*** | 0.03 | 0.03 | 8.41 | 1.60 | -1.25 | 1.06 |
| ***cdgH*** | 0.02 | 0.01 | 7.15 | 0.76 | -1.34 | 0.04 |
| ***vpvC*** | 0.04 | 0.04 | 6.71 | 0.54 | -0.99 | 0.36 |
| ***cdpA*** | 0.32 | 0.33 | 5.59 | 0.22 | -0.28 | 0.15 |

 Transcript levels of c‑di‑GMP‑related regulatory genes in the WT, *ΔdegS*, and complemented strain (*ΔdegS::degS*) cultured in M9 medium. Data are presented as mean ± SD from three independent biological replicates (n=3). The relative expression of each gene was calculated using the 2^⁻ΔΔCt^ method, with 16S rRNA as the internal control and the WT strain as the calibrator (set to 1). Data are presented on a log₂ scale.

**Figure 5G**

|  | **WT** | | ***ΔdegS*** | | ***ΔdegSΔcdgH*** | | ***ΔdegSΔcdgH::cdgH*** | | ***ΔdegSΔcdgH+pBAD24*** | |
| --- | --- | --- | --- | --- | --- | --- | --- | --- | --- | --- |
| **Gene** | **Mean** | **SD** | **Mean** | **SD** | **Mean** | **SD** | **Mean** | **SD** | **Mean** | **SD** |
| ***vpsR*** | 1.13 | 0.13 | 32.47 | 6.59 | 3.09 | 0.12 | 37.97 | 11.66 | 4.14 | 0.36 |

*vpsR* transcript levels in the WT, *ΔdegS*, *ΔdegSΔcdgH*, *ΔdegSΔcdgH::cdgH*, and *ΔdegSΔcdgH* + pBAD24 strains cultured in M9 medium. Data are presented as mean ± SD from three independent biological replicates (n=3). The relative expression of each gene was calculated using the 2^⁻ΔΔCt^ method, with 16S rRNA as the internal control and the WT strain as the calibrator (set to 1).
